# Mental health of UK youth following the removal of the Education Maintenance Allowance in England: a natural experiment study using Understanding Society data

**DOI:** 10.1136/bmjph-2024-001677

**Published:** 2025-06-03

**Authors:** Richard John Shaw, Andrew James Baxter, Srinivasa Vittal Katikireddi

**Affiliations:** 1 MRC/CSO Social and Public Health Sciences Unit, School of Health and Wellbeing, University of Glasgow, Glasgow, UK

**Keywords:** Education, Mental Health, Sociodemographic Factors, trends, statistics and numerical data

## Abstract

**Background:**

The Education Maintenance Allowance (EMA) encourages people in low-income households to stay in education. It was abolished in England but continues in the rest of the UK (RUK). We investigated if the abolition of the EMA was associated with psychological distress using a difference-in-difference design.

**Methods:**

The sample of 1328 observations was drawn from Understanding Society participants aged 16/17 at the start of the academic years 2009–2018 and in the bottom income decile. Exposure to the EMA policy regime was identified using an interaction between the UK area (RUK vs England) and EMA policy period. The 2009/2010 academic years indicated the EMA period, 2011 the transition period (when EMA receipt was limited to existing recipients) and 2012–2018 post-EMA. The primary outcome was the General Health Questionnaire-12 (GHQ-12) score ranging 0–36. Other outcomes include the 12-item Short-Form Health Survey (SF-12) and a physical health falsification outcome. Linear regression using robust SEs, adjusting for sex and month of interview, was conducted.

**Results:**

In England, relative to RUK and the EMA period, the transition period (Coef 4.20; 95% CI 1.12 to 7.28) and the post-EMA period (Coef 2.89; 95% CI 0.67 to 5.11) were associated with worse GHQ-12 scores. Results for other mental health outcomes were similar, with no associations with the falsification outcome.

**Conclusions:**

Young people living in low-income households in England appeared to have worse mental health following the removal of the EMA, compared with RUK. However, it is not possible to rule out the potential contribution of cointerventions, such as the raising of mandatory age for education to 17 years in 2013 and 18 years in 2015.

**WHAT IS ALREADY KNOWN ON THIS TOPIC**

- The Education Maintenance Allowance (EMA) increased the proportion of people staying on in education beyond school leaving age.
- How the EMA influences the mental well-being of young people in low-income households is unknown.

**WHAT THIS STUDY ADDS**

- Abolition of the EMA in England was associated with increased psychological distress among young people in low-income households, although the peak coincided with raising the mandatory education age to 17.

**HOW THIS STUDY MIGHT AFFECT RESEARCH, PRACTICE OR POLICY**

- The possibility that the EMA may have fewer adverse consequences when compared with other means of encouraging continued education, such as laws mandating participation in education, needs to be considered when developing policy.

## Introduction

 Common mental disorders are among the top 10 leading causes of disability globally,^1^ and psychological distress was increasing even before the COVID-19 pandemic.^2^ The UK is no exception,^3^ with the greatest increases in psychological distress among the young and socio-economically disadvantaged. Adolescence is a critical period for development, with poor mental health being linked to future unemployment and poor health.^4^ Identifying policies which might improve young disadvantaged people’s mental health is essential, and education is a policy area that is susceptible to intervention.^5^ People who leave education and fail to find subsequent employment or training—otherwise termed Not in Education Employment or Training (NEET)—are at particularly high risk for poor mental health.^6 7^ Reducing the number of adolescents who are considered NEET has been a priority for many UK and other governments. The Education Maintenance Allowance (EMA) is a policy that has been successful in improving educational outcomes,^8^ but little is known about the EMA’s impact on mental health.

The EMA is a conditional cash transfer programme providing a weekly stipend of up to £30 to people on condition that they are 16–19 years old, receive appropriate education or training and have household incomes below a threshold.^9 10^ For England, the income threshold for receiving the maximum amount was £20 817 (approximately $32 000 dollars in 2010),^10^ with minor variations across UK nations, year and household size. After piloting, the EMA was introduced UK wide in 2004 and it increased the proportion staying in education by 7.4 percentage points for eligible males and 5.9 percentage points for eligible females.^9^ It is estimated that two-thirds of those who stayed in education as a result of the EMA would have been economically inactive.^9^

UK education policy is devolved, when the UK government changed in 2010, each of the UK’s national governments made decisions relating to the EMA.^10^,^12^ England abolished the EMA during the 2010/2011 school year, the replacement being a bursary fund targeted mainly at those in care or care leavers.^11^ A transitional phase for the 2011 school year continued the EMA for pre-existing EMA recipients. The rest of the UK (RUK—Scotland, Wales and Northern Ireland) maintained the EMA, but targeted resources at the most disadvantaged households meeting the threshold for the full £30 stipend. Additionally, a change to English law, raising the mandatory age for education or training until the child turned 17 years in 2013 and 18 years in 2015, was used to justify the abolition of the EMA,^13^ and created a partly overlapping cointervention. Using the divergence of policies between countries, we aim to estimate the effect of abolishing the EMA on mental health by treating countries as exposed and unexposed units in a natural experiment. If the EMA policy influences mental health, it would be expected that education, employment status and financial strain would account for some of the EMA’s associations. Our primary research question evaluating the EMA is:

RQ1: Among young people in low-income households, does the difference in average mental health between people in England and RUK change when comparing the transitional and post-EMA periods to the period prior to the abolition of EMA in England?

To explore the possible mechanism through which the EMA operates, we ask:

RQ2: Does adjusting for educational status and financial strain reduce associations between EMA policy period and mental health?

To investigate the influence of possible cointerventions, we ask:

RQ3: Do any observed differences seen in RQ1 remain when the post-EMA period is stratified to account for the period when the cointervention of raising compulsory education age in England occurred?

## Methods

### Study design

The impact of the abolition of the EMA in England for adolescents aged 16 or 17 living in households in the bottom 10% of household income was assessed with an intention-to-treat difference-in-differences design. Our estimates of the difference-in-differences were calculated using an interaction between UK country of residence at the time of the interview and EMA policy period. The UK country of residence was defined as RUK versus England. In the primary analyses EMA period was defined by allocating academic years as follows: the 2009 and 2010 academic years as the EMA period, 2011 as a transition year, and 2012–2018 as the post-EMA period.

### Pre-registration

The study protocol was pre-registered on Open Science Framework (https://osf.io/u3avm), with any deviations detailed in online supplemental file.

### Public involvement

No members of the public were involved in the design, conduct or reporting of these analyses.

### Data source

Understanding Society is a nationally representative household panel study based on a stratified random probability sample of around 40 000 households.^14^ Data are collected from all adults (aged≥16) in participating households by interviewers. Each wave of data collection lasts 24 months, but participants are followed up annually, with interviews scheduled at the same time each year. We adapted the pre-existing cross-sectional weights for the self-completion questionnaire so that they could be applied for the academic years (running from 1 September to 31 August) from 2009 to 2018 rather than the study waves normally used for analysis. (Full details of code are available from https://github.com/rickwahs/EMA-USoc & https://osf.io/u3avm.)

### Study population

The unit of analysis is participants’ observations for each academic year selected as follows (see online supplemental figure 1 for n at each stage): (1) had a valid academic year weight; (2) were aged 16 or 17 on 1 September of that academic year (to ensure eligible for receipt of EMA); (3) were resident in England, Scotland, Wales or Northern Ireland; (4) were not living with foster parents, (people living with foster parents are likely to be eligible for the bursary scheme—a cointervention); (5) were from an interview response during the 2009–2018 academic years; (6) had a valid response for any of the outcome measures; (7) were estimated to be in the bottom 10% of gross household incomes for that academic year derived from responses to the household questionnaire. We selected the bottom 10% to ensure a consistent level of social disadvantage which is unlikely to exceed the income eligibility threshold for receipt of EMA across time.

### Outcome

The primary outcome was the General Health Questionnaire-12 (GHQ-12) score, calculated using continuous Likert scoring ranging from 0 to 36. Higher scores indicating psychological distress.^15^ Additional mental health measures, for which lower scores indicate worse mental health, are: the Mental Component Summary (MCS) of the 12-item Short-Form Health Survey (SF-12) which assesses mental health, social functioning and emotional problems,^16^ and life satisfaction based on a 7-point Likert scale (completely dissatisfied to completely satisfied). As a falsification outcome, we included the Physical Component Summary (PCS) of the SF-12, which assesses physical functioning^16^ for which we did not expect associations due to the likely time lag before impacts would arise.

### Statistical analysis

To estimate the difference-in-differences for the EMA policy periods in England relative to RUK, we created sets of models including an interaction term between UK country and EMA period to predict each of the outcomes. We created these models using linear regression with robust SEs accounting for academic year weights using lm_robust in R. The equations for models 1, 2, and 3 are included in the online supplemental file. In model 1 addressing RQ1, we adjusted for participants’ age at the start of the academic year, sex and month of interview.

To address RQ2, we first created model 2, which was based on model 1 and additionally adjusted for financial strain and education/employment status, which were assessed using self-reported measures for the individual. Model 3 was based on model 2 with additional covariates to improve model precision and close any back door pathways^17^ introduced by financial strain and education. Additional covariates (categories shown in table 1) in model 3 were: housing tenure, number of children under 15 in household, two parent household, working parent, highest reported parental educational qualification, ethnicity and UK country.

**Table 1:** Number and per cent for the categorical variables used in analyses by EMA policy period for young people living in households in the bottom 10% of household incomes

	EMA period2009 and 2010		Transition2011		Post-EMA2012–2018		Total	
	n	%	n	%	n	%	n	%
Area of the UK								
RUK	84	25.4	45	29.0	147	17.5	276	20.8
England	247	74.6	110	71.0	695	82.5	1052	79.2
Sex								
Male	155	46.8	76	49.0	391	46.4	622	46.8
Female	176	53.2	79	51.0	451	53.6	706	53.2
Age at start of school year								
16	169	51.1	77	49.7	411	48.8	657	49.5
17	162	48.9	78	50.3	431	51.2	671	50.5
Financial strain								
Comfortable	52	15.8	25	16.2	213	25.8	290	22.2
Alright	135	41.0	51	33.1	338	40.0	524	40.0
Getting by	81	24.6	51	33.1	188	22.8	320	24.4
Quite difficult	39	11.9	18	11.7	60	7.3	117	8.9
Very difficult	22	6.7	9	5.8	27	3.3	58	4.4
Missing	2		1		16		19	
Education status								
In education	221	66.8	100	64.5	666	79.1	987	74.3
Emp and Train	28	8.5	13	8.4	68	8.1	109	8.2
NEET	82	24.8	42	27.1	108	12.8	232	17.5
Household tenure								
Owners	107	32.4	54	35.3	359	43.1	520	39.5
Rent and other	223	67.6	99	64.7	474	56.9	796	60.5
Missing	1		2	0.0	9		12	
Number of other children								
None	196	59.2	85	54.8	441	52.4	722	54.4
One or two	116	35.0	62	40.0	344	40.9	522	39.3
Three or more	19	5.7	8	5.2	57	6.8	84	6.3
Two parents in household								
No	250	75.5	123	79.4	586	69.6	959	72.2
Yes	81	24.5	32	20.6	256	30.4	369	27.8
Parent working								
Working parent	106	32.0	61	39.4	426	50.6	593	44.7
No working parent	225	68.0	94	60.6	416	49.4	735	55.3
Highest parental ed								
Post-school	58	17.5	27	17.4	222	26.4	307	23.1
School level	105	31.7	66	42.6	340	40.4	511	38.5
No or other	123	37.2	46	29.7	192	22.8	361	27.2
Other not classifiable	45	13.6	16	10.3	88	10.5	149	11.2
Ethnicity								
White British	201	64.0	94	67.6	488	58.8	783	61.0
Other	113	36.0	45	32.4	342	41.2	500	39.0
Missing	17		16		12		45	
Detailed country								
England	247	74.6	110	71.0	695	82.5	1052	79.2
Wales	25	7.6	12	7.7	52	6.2	89	6.7
Scotland	27	8.2	17	11.0	44	5.2	88	6.6
Northern Ireland	32	9.7	16	10.3	51	6.1	99	7.5

EMA, Education Maintenance Allowance; NEET, Not in Education Employment or Training; RUK, rest of the UK.

To address RQ3, we created model 4 using a variable that subdivides the EMA period, accounting for the increases in compulsory education age. The academic years were classified as follows: 2009 and 2010 (the EMA period); 2011 (transition year), 2012 (post-EMA period), 2013 and 2014 compulsory education until child turns 17 (age 17), and 2015–2018 compulsory education until child turns 18 (age 18).

### Sensitivity analyses

In sensitivity analyses, we changed the analytic samples for model 1 as follows:

Sensitivity 1: widened the sample to the bottom 15% of household income.

Sensitivity 2: changed the analytic sample to the top 80% of incomes, who were unlikely to be eligible to receive the EMA.

Sensitivity 3: only included observations for whom less than 10% of household income data was imputed by Understanding Society.

Sensitivity 4: investigated the sensitivity of the models to using weights by re-running the analyses using the original cross-sectional self-completion weights.

We also carried out an additional falsification test by restricting the sample to young people in England and contrasted the life-satisfaction measure for 16/17-year-olds with the most similar available measure for 13/14-year-olds who completed the youth questionnaire, which was a measure of overall happiness.

Finally, we ran analyses stratified by gender, ethnicity and age at start of school year, and using a square root transformed GHQ measure.

Survey non-response is accounted for by using weighting and missing data for the covariates are handled using complete case analysis. Analyses were carried out using Stata V.17.0 and R V.4.3.2 with code available online (https://github.com/rickwahs/EMA-USoc).

## Results

### Descriptives

The number and unweighted percentages for categorical variables are shown in table 1. Table 2 shows the analytic sample means and SD for the outcomes. For GHQ-12, MCS and life satisfaction, there is a general pattern of deteriorating mental well-being in England. The RUK has somewhat worse health than in England in the initial EMA period, but improves somewhat over time. There is a modest increase in PCS (indicating a possible slight improvement in health) for England, while there is a slightly greater improvement for the RUK.

**Table 2:** Mean and SD for GHQ-12, SF-12 Mental and Physical Component Scales, and life satisfaction for young people (aged 16 or 17) at the start of the academic year living in households in the lowest 10% of income, by policy period and area of UK

Policy period (academic year)	GHQ-12		MCS		PCS		Life satisfaction	
	Mean	SD	Mean	SD	Mean	SD	Mean	SD
Rest of UK								
EMA period (2009 and 2010)	12.0	6.9	48.2	11.6	52.1	9.1	5.2	1.7
Transition (2011)	9.8	5.8	51.6	8.0	51.6	7.8	5.5	1.5
Post-EMA (2012–2018)	10.6	5.4	47.9	11.5	55.1	7.5	5.4	1.5
England only								
EMA period (2009 and 2010)	9.6	5.4	50.7	9.9	53.8	6.6	5.5	1.4
Transition (2011)	12.0	6.0	45.7	11.5	54.9	5.9	5.0	1.6
Post-EMA (2012–2018)	11.4	6.3	46.0	12.7	54.5	7.0	5.2	1.6
All of UK								
EMA period (2009 and 2010)	10.1	5.8	50.2	10.3	53.5	7.2	5.4	1.5
Transition (2011)	11.5	6.0	47.0	11.0	54.2	6.5	5.1	1.6
Post-EMA (2012–2018)	11.3	6.2	46.3	12.5	54.6	7.1	5.2	1.6

EMA, Education Maintenance Allowance; GHQ-12, General Health Questionnaire-12; MCS, Mental Component Summary; PCS, Physical Component Summary; SF-12, 12-item Short-Form Health Survey.

### Regression analyses RQs 1 and 2

#### Primary outcome—GHQ-12

The main difference-in-differences estimates for GHQ-12 for models 1, 2 and 3 are shown in table 3 (all regression coefficients are in online supplemental table 1). For model 1, which addresses RQ1, during the transition period (Coef 4.20, 95% CI 1.12 to 7.28) and post-EMA period (Coef 2.89, 95% CI 0.67 to 5.11), youth in England fared worse than those in RUK. (Online supplemental figure 2 shows predicted mental health outcomes by EMA policy period from model 1.) In model 2, which adjusts for both financial strain and education and employment status to investigate if they might explain the difference-in-differences estimates (RQ2), the associations become slightly stronger during both the transition period (Coef 4.77, 95% CI 1.75 to 7.80) and post-EMA period (Coef 3.16, 95% CI 0.92 to 5.40). Model 3, which adjusts for additional covariates to block possible back door pathways opened by model 2, again shows an increased risk of psychological distress in both the transition period (Coef 5.01, 95% CI 1.80 to 8.22) and the post-EMA period (Coef 3.09, 95% CI 0.89 to 5.29) in England.

**Table 3:** Difference-in-differences† estimates for the associations with changing EMA policy period in England on GHQ-12, MCS, PCS and life satisfaction after minimal adjustment model 1, adjusting for financial strain and educational status model 2 and adjusting for all covariates model 3‡ for young people in the lowest 10% of household incomes

Reference period 2009 and 2010	Model 1§	Model 2¶	Model 3††
	Coef (95% CI)	Coef (95% CI)	Coef (95% CI)
GHQ-12			
Transition period (2011)	4.20 (1.12 to 7.28)**	4.77 (1.75 to 7.80)**	5.01 (1.80 to 8.22)**
Post-EMA (2012–2018)	2.89 (0.67 to 5.11)*	3.16 (0.92 to 5.40)**	3.09 (0.89 to 5.29)**
MCS			
Transition period (2011)	−7.45 (−12.27 to −2.64)**	−8.64 (−13.34 to −3.94)***	−8.06 (−12.80 to −3.31)***
Post-EMA (2012–2018)	−3.79 (−7.95 to 0.37)+	−4.37 (−8.60 to −0.14)*	−3.59 (−7.63 to 0.44)+
PCS			
Transition period (2011)	2.04 (−1.80 to 5.88)	1.26 (−2.45 to 4.96)	0.81 (−2.96 to 4.58)
Post-EMA (2012–2018)	−2.13 (−5.09 to 0.83)	−1.82 (−4.71 to 1.07)	−1.84 (−4.72 to 1.05)
Life satisfaction			
Transition period (2011)	−0.81 (−1.65 to 0.03)+	−0.99 (−1.74 to −0.23)*	−0.75 (−1.54 to 0.04)+
Post-EMA (2012–2018)	−0.47 (−1.03 to 0.08)+	−0.55 (−1.11 to 0.02)+	−0.42 (−0.97 to 0.13)

*p≤0.05, **p≤0.01, ***p≤0.001, +p≤0.1.

†An interaction term between UK country and EMA period.

‡Full models showing all regression coefficients are shown in online supplemental table 1 (GHQ-12), online supplemental table 2 (-MCS), online supplemental table 3 (-PCS) and online supplemental table 4 (life satisfaction).

§Model 1 includes main effects for UK area, EMA policy period, age at start of school year, gender, month of interview as well as difference-in-differences estimate.

¶Model 2 in addition to the variables in model 1, this model adds financial strain, and education status.

††Model 3 in addition to the variables in model 2 adds two parent household, working parent, housing tenure, other children in household, parental education, ethnicity and additional dummy variables indicating country.

EMA, Education Maintenance Allowance; GHQ-12, General Health Questionnaire-12; MCS, Mental Component Summary; PCS, Physical Component Summary.

#### Secondary outcomes—SF-12 MCS and life satisfaction

The results for difference-in-differences analyses for MCS and life satisfaction for models 1, 2 and 3 are shown in table 3 (all the regression coefficients for these models are shown in online supplemental tables 2 and 3). The MCS results are consistent with those for the GHQ-12: again, there is a stronger adverse association in the transition period (Coef −7.45, 95% CI −12.27 to −2.64) than the post-EMA period (Coef −3.79 95% CI −7.95 to 0.037), and the increased adjustment in models 2 and 3 makes little difference. Similarly, results in table 3 show relative falls in life satisfaction during the transition period (Coef −0.91, 95% CI −1.65 to 0.03) and the post-EMA period (Coef −0.47, 95% CI −1.03 to 0.08) in models 1, 2 and 3 (see table 3).

#### Falsification outcome

For PCS, there is little evidence of an association for the difference-in-differences estimates in either the transition period (Coef 2.04, 95% CI −1.80 to 5.88), or the post-EMA period (Coef −2.13, 95% CI −5.09 to 0.83) in model 1. Effect sizes changed little on further adjustment in models 2 and 3 (see table 3 and online supplemental table 4).

### Regression analyses RQ3

In the analyses subdividing the EMA period to account for changes in school leaving age, the greatest increase in GHQ-12 was when school leaving age was raised to 17 in 2013 and 2014 (Coef 5.19, 95% CI 2.64 to 7.75), and not the period when the EMA was first abolished in 2012 (Coef 1.28, 95% CI −2.16 to 4.73) or the later subsequent periods when school leaving age was increased further to age 18 in 2015 (Coef 1.87, 95% CI −0.71 to 4.44). Similar results were found for both MCS and life satisfaction (table 4), and there was little evidence of any associations with PCS.

**Table 4:** Difference-in-differences estimates† for the associations with changing EMA policy period in England on GHQ-12, MCS, PCS and life satisfaction using subdividing the post-EMA policy period to account for school leaving age increasing (model 4) for young people in the lowest 10% of household incomes

Policy period (academic years)	GHQ-12	MCS	PCS	Life satisfaction
	Coef (95% CI)	Coef (95% CI)	Coef (95% CI)	Coef (95% CI)
EMA period (Ref—2009 and 2010)				
Transition period (2011)	4.20 (1.12 to 7.28)**	−7.44 (−12.26 to −2.61)**	2.02 (−1.82 to 5.85)	−0.81 (−1.65 to 0.03)+
Post-EMA (2012)	1.28 (−2.16 to 4.73)	−3.02 (−8.61 to 2.55)	−0.60 (−4.65 to 3.45)	0.18 (−0.78 to 1.14)
School leaving age 17 (2013–2014)	5.19 (2.64 to 7.75)***	−7.98 (−12.64 to −3.32)***	−0.85 (−4.25 to 2.55)	−1.09 (−1.71 to −0.47)***
School leaving age 18 (2015–2018)	1.87 (−0.71 to 4.44)	−1.15 (−6.48 to 4.18)	−3.36 (−6.99 to 0.27)+	−0.24 (−0.89 to 0.42)

* p≤0.05, **p≤0.01, ***p≤0.001, +p ≤0.1.

† An interaction term between UK country and EMA period. Models also include main effects for UK area, EMA policy period, age at start of school year, gender, month of interview as well as the difference-in-differences estimate.

EMA, Education Maintenance Allowance; GHQ-12, General Health Questionnaire-12; MCS, Mental Component Summary; PCS, Physical Component Summary.

### Sensitivity analyses

Broadening the sample to include those in the bottom 15% (sensitivity 1 in table 5, n=1946) led to relatively small reductions in all the associations. Repeating the analyses with people in the top 80% of household income (sensitivity 2, n=9165) led to much smaller coefficients consistent with a null effect, except for the falsification outcome, PCS, for which there was a small relative decline (Coef −0.88, 95% CI −1.66 to −0.09). Excluding participants with imputed income data (sensitivity 3) reduced the overall sample size considerably (n=778), with slightly increased associations for the post-EMA period for GHQ-12 and MCS, and reduced associations for the transition period. Using the original self-completion weights based on wave, rather than those based on academic year (sensitivity 4), had little impact on results. There was little evidence of variations within any of the strata including: gender (online supplemental table 5), ethnicity (online supplemental table 6) and age (online supplemental table 7), although CIs were quite wide. In analyses (n=2025) contrasting 16/17 year olds (life-satisfaction) with 13/14 years olds (happiness), we found a weak non-significant deterioration during the transition period (Coef −0.34, 95% CI –0.85 to 0.18) for 16/17 year olds relative to 13/14 year olds, and little difference in the post-EMA period (Coef −0.05, 95% CI −0.37 to 0.26). When GHQ-12 was square root transformed (transition period: Coef 0.70, 95% CI 0.23 to 1.17; post-EMA period: Coef 0.41, 95% CI 0.09 to 0.73) results were consistent with the original measure.

**Table 5:** Difference-in-difference estimates† for the associations with changing EMA policy period in England on GHQ-12, MCS, PCS and life satisfaction after minimal adjustment (model 1), and sensitivity analyses expanding the sample to participants in the bottom 15% (sensitivity 1) and changing to the top 80% of household incomes (sensitivity 2), excluding those with imputed household income data (sensitivity 3) and using the original cross-sectional sample weights (sensitivity 4)

Reference period 2009 and 2010	Original analyses	Sensitivity 1: bottom 15% of income	Sensitivity 2: top 80% of income	Sensitivity 3: sample excluding imputed income data	Sensitivity 4: sample using original weights
	Coef (95% CI)	Coef (95% CI)	Coef (95% CI)	Coef (95% CI)	Coef (95% CI)
GHQ-12					
Transition period (2011)	4.20 (1.12 to 7.28)**	3.28 (0.87 to 5.69)**	−0.83 (−1.89 to 1.78)+	2.45 (−1.35 to 6.24)	3.93 (0.94 to 6.93)**
Post-EMA (2012–2018)	2.89 (0.67 to 5.11)*	1.58 (−0.19 to 3.35)+	−0.37 (−0.98 to 0.24)	3.17 (0.48 to 5.87)*	2.75 (0.61 to 4.88)**
MCS					
Transition period (2011)	−7.45 (−12.27 to −2.64)**	−5.84 (−9.98 to −1.69)**	1.32 (−0.31 to 2.96)	−6.70 (−12.40 to −1.00)*	−7.30 (−12.06 to −2.55)**
Post-EMA (2012–2018)	−3.79 (−7.95 to 0.37)+	−2.59 (−5.94 to 0.76)	0.89 (−0.25 to 2.04)	−4.54 (−9.84 to 0.77)+	−3.95 (−8.01 to 0.12)+
PCS					
Transition period (2011)	2.04 (−1.80 to 5.88)	1.85 (−1.49 to 5.20)	−0.38 (−1.56 to 0.80)	2.09 (−2.61 to 6.79)	2.21 (−1.62 to 6.03)
Post-EMA (2012–2018)	−2.13 (−5.09 to 0.83)	−0.96 (−3.35 to 1.44)	−0.88 (−1.66 to −0.09)*	−2.47 (−6.11 to 1.17)	−1.96 (−4.88 to 0.95)
Life satisfaction					
Transition period (2011)	−0.81 (−1.65 to 0.03)+	−0.66 (−1.30 to −0.01)*	0.26 (−0.04 to 0.56)+	−0.84 (−1.70 to 0.02)+	−0.83 (−1.65 to 0.00)*
Post-EMA (2012–2018)	−0.47 (−1.03 to 0.08)+	−0.26 (−0.71 to 0.19)	0.14 (−0.05 to 0.32)	−0.46 (−1.16 to 0.23)	−0.48 (−1.03 to 0.06)+

* p≤0.05, **p≤0.01, ***p≤0.001, +p ≤0.1.

† An interaction term between UK country and EMA period. Models also include main effects for UK area, EMA policy period, age at start of school year, gender, month of interview, as well as the difference-in-difference estimate.

EMA, Education Maintenance Allowance; GHQ-12, General Health Questionnaire-12; MCS, Mental Component Summary; PCS, Physical Component Summary.

## Discussion

During the transition and post-EMA periods, the mental health of young people in England’s most disadvantaged households deteriorated relative to equivalent people in RUK. We did not find a deterioration in physical health as indicated by the PCS, nor did we find such changes in mental health among young people in more advantaged households not eligible for the EMA. These falsification tests indicate that there is not a common cause affecting both the exposed and unexposed, nor a longer-term process affecting both physical and mental health. However, it is questionable if the EMA alone is the main driver of the mental health changes. The difference-in-differences was not reduced on adjustment for financial strain, educational attainment and employment status. We find the strongest associations for the transitionary period, rather than the post-EMA period. Within the post-EMA period, the strongest associations were seen in the period when compulsory education was raised to 17. In addition, within England, 16/17 year-olds do not do significantly worse than 13/14 year-olds. Overall, the changes may be consistent with more general consequences arising from austerity^18^ or educational policy changes.

EMA had a positive impact on continued education,^8^ and education is considered an important risk factor for mental health.^5^ However, we are only aware of one working paper^19^ that investigated the relationship between the EMA and mental health. Using inverse probability weighted regression analyses, it was found that receipt of EMA was associated with educational and employment outcomes, but not GHQ-12, among a general population cohort study who were self-selecting for receipt of EMA, which may have biased results.

Our results show a worsening of young disadvantaged people’s mental health in England. However, if education is always beneficial for mental health, it might be expected that the association would be strongest when the EMA was abolished, but before compulsory education was extended, which was not found. More research is needed on possible heterogeneity in the relationship between education and psychological distress.^20 21^ Short-term adverse consequences of continuing in education are possible for young people who feel they do not benefit from education.^22^ For some, continuing in education may result in opportunity costs or prolonging the stresses of a competitive school environment.^22^ Evidence is currently limited, and studies are context specific. UK studies focusing on the increases in school leaving age in 1947 and 1972^23^ suggest either a neutral^23^ or negative^22^ influence of extending education on later mental health. The EMA, which uses positive incentives to encourage education, may offer more benefits and fewer adverse consequences than extending compulsory education.

A general deterioration of mental health in the post-EMA period is more consistent with the loss of the financial stipend introduced by the EMA. If this is the pathway through which the EMA operates, we would have expected the difference-difference coefficient to reduce when financial strain was introduced in model 2, which we did not find. Adolescent mental health has deteriorated in the context of rising precarity, erosion of job security for young people, increasing debts and worsening prospects of important life transitions such as home ownership.^24^ Poor adolescent mental health now accounts for at least 45% of the overall burden of disease for young people.^24^ In the UK, those from lower-income households who would be eligible for the EMA have borne the brunt of these changes. In any case, given that the EMA continues in the RUK, income thresholds for eligibility and stipend levels will need to be revised. Initiatives should be put in place to ensure such policy changes are implemented in ways that make their evaluation easier. This could include routine collection of indicators of well-being and implementing policies in ways that enable causal inference using weaker assumptions.

### Strengths and limitations

A strength is the use of an intention-to-treat design to investigate relationships at population level, avoiding self-selection resulting from receipt of EMA. However, there are potential cointerventions alongside the EMA, which may have mitigated the impact of the abolition of EMA. The most direct replacement for the EMA, the bursary fund was mainly targeted at those in care or care leavers,^11^ who were excluded from our analytic sample. Another limitation is that Understanding Society did not collect data before 2009 nor collect the same measures for people under the age of 16. This limited the ability to adjust for participants’ prior health, and the ability to investigate the parallel trends assumption using Understanding Society data alone. As an alternative, we aimed to investigate the plausibility of parallel trends using BHPS data from 2001 and 2008. These data show youth in the RUK have worse GHQ-12 scores (see online supplemental figure 3). Unfortunately, the number of observations (n=687) for young people in low-income households was low, there are fluctuations which are within the boundaries of chance. So, it is unclear how well the parallel trends assumption holds. Finally, while we have the power to identify clear differences between mental health before and after the abolition of the EMA, the sample size is relatively small and the CIs post-EMA were quite wide, giving imprecise estimates of any changes when the cointerventions were introduced.

## Conclusions

Young people in England in the bottom 10% of household incomes when compared with RUK had higher psychological distress following the abolition of EMA in England. However, the highest levels of psychological distress coincide with increasing the compulsory education age to 17, and the lack of explanatory effects by financial strain and education and employment status suggests alternative explanations. The EMA continues in the RUK and will need reviewing to ensure that stipend levels and income thresholds remain relevant. Any possible policy changes should be evaluated taking into account health as well as educational outcomes. The EMA may have less adverse consequences than other means of encouraging continued education.

## Supplementary material

10.1136/bmjph-2024-001677 online supplemental file 1

## Data Availability

Data are available in a public, open access repository.
