# Supplementary material for: Mental health of UK youth following the removal of the Education Maintenance Allowance in England: a natural experiment study using Understanding Society data
Source: BMJ Public Health. 2025 Jun 3;3(1):e001677. doi: 10.1136/bmjph-2024-001677 (PMC12164303; doi:10.1136/bmjph-2024-001677)

# Supplementary File

## Deviations from the study protocol

Deviations from the study protocol pre-registered on Open Science Framework (<https://osf.io/u3avm>) are as follows:

We omitted two sensitivity analyses. There were very few observations with negative incomes which might influence results, thus re-running analyses omitting these people is unlikely to have had an impact. We also did not repeat analyses including people without valid weights. The latter sensitivity analyses would have been to estimate how much impact weighting had on the results. However, it inevitably would have introduced biases. We consider sensitivity analysis four a suitable alternative. Sensitivity analysis four reran analyses using the original cross-sectional self-completion weight.

Sensitivity 2 in which we changed the analytic sample to those in the top 80% of household income was an additional analysis not included in the original study protocol.

We added an additional falsification test by restricting the sample to young people in England and contrasted the life-satisfaction measure for 16/17 year olds with a measure of a happiness for 13/14 year olds.

## Regression equations

### Model 1

$$y_{it}=\alpha+{\gamma Country}_{it} +{\lambda Period}_{it}+ \delta\left( {Country}_{it} \cdot{Period}_{it} \right)+ P_{it}^{'}\beta_{1}+ \varepsilon_{it}$$

Where:

y is the health outcome.

i is individual

t is the wave in which a person responded

α is the expected value for the for the reference category.

γ is the coefficient for the *Country* variable (The rest of the UK versus England)

λ is the coefficient for the *Period* variable (EMA period versus England)

δ is the coefficient for the Difference-in-differences, the interaction term between *Country* and *Period*

P_it_ is a vector of covariates (age at start of school year, sex, and month of interview) for individual i in survey wave t. β_1_ is a vector of corresponding coefficients.

ε is the error term.

### Model 2

$$y_{it}=\alpha+{\gamma Country}_{it} +{\lambda Period}_{it}+ \delta\left( {Country}_{it}\cdot{Period}_{it} \right)+P_{it}^{'}\beta_{1}+Q_{it}^{'}\beta_{2}+\varepsilon_{it}$$

Model 2 adds Q a vector of variables that might act as mechanisms (financial strain and education) for the EMA. β_2_ is a vector of the corresponding regression coefficients.

### Model 3

$$y_{it}=\alpha+{\gamma Country}_{it}+{\lambda Period}_{it}+\delta\left( {Country}_{it}\cdot{Period}_{it} \right)+P_{it}^{'}\beta_{1}+Q_{it}^{'}\beta_{2}+R_{it}^{'}\beta_{3}+\varepsilon_{it}$$

Model 3 adds R a vector of variables (housing tenure, number of children under 15 in the household, two parent household, working parent, highest reported parental qualification, ethnicity and UK country) to close back door pathways introduced in model 2.

## Tables

Supplementary table 1: All regression coefficients for models 1, 2, 3, predicting GHQ-12 for young people in the bottom 10% of incomes in Understanding Society.

| **Variable (ref category)** | Model 1 | Model 2 | Model 3 |
| --- | --- | --- | --- |
|  | Coef (95% CI) | Coef (95% CI) | Coef (95% CI) |
| Intercept | 10.23 ( 8.09 to 12.37) | 8.86 ( 6.56 to 11.17) | 9.75 ( 7.09 to 12.41) |
| Area of UK - RUK |  |  |  |
| England | -2.21 (-4.10 to -0.33) | -2.23 (-4.13 to -0.33) | -2.45 (-4.68 to -0.22) |
| *EMA Policy Period (EMA Period)* |  |  |  |
| Transition | -1.82 (-4.50 to 0.86) | -2.18 (-4.85 to 0.49) | -1.96 (-4.75 to 0.83) |
| Post EMA | -1.17 (-3.18 to 0.85) | -1.05 (-3.10 to 0.99) | -0.82 (-2.83 to 1.18) |
| *Age start school year (16)* |  |  |  |
| 17 | 0.59 (-0.21 to 1.39) | 0.39 (-0.41 to 1.19) | 0.44 (-0.38 to 1.27) |
| *Gender (Male)* |  |  |  |
| Female | 1.90 ( 1.10 to 2.70) | 2.14 ( 1.34 to 2.93) | 2.22 ( 1.40 to 3.04) |
| *Month of interview (January)* |  |  |  |
| February | 0.27 (-1.36 to 1.90) | 0.66 (-0.99 to 2.31) | 0.68 (-1.01 to 2.36) |
| March | 0.07 (-1.82 to 1.97) | 0.38 (-1.58 to 2.34) | 0.63 (-1.46 to 2.71) |
| April | 0.93 (-0.67 to 2.53) | 1.02 (-0.65 to 2.70) | 0.92 (-0.83 to 2.68) |
| May | 1.37 (-0.45 to 3.20) | 1.17 (-0.73 to 3.07) | 1.11 (-0.84 to 3.07) |
| June | -0.15 (-1.85 to 1.55) | -0.07 (-1.82 to 1.69) | -0.08 (-1.89 to 1.73) |
| July | 1.10 (-0.88 to 3.09) | 1.35 (-0.68 to 3.39) | 1.17 (-0.86 to 3.20) |
| August | 0.90 (-1.00 to 2.80) | 1.27 (-0.66 to 3.20) | 1.26 (-0.79 to 3.31) |
| September | -0.25 (-2.02 to 1.53) | 0.17 (-1.63 to 1.97) | 0.21 (-1.62 to 2.04) |
| October | -1.58 (-2.99 to -0.17) | -1.46 (-2.89 to -0.02) | -1.44 (-2.97 to 0.09) |
| November | 0.28 (-1.38 to 1.94) | 0.36 (-1.40 to 2.12) | 0.65 (-1.12 to 2.42) |
| December | 1.92 (-0.22 to 4.06) | 2.33 ( 0.18 to 4.48) | 2.44 ( 0.14 to 4.74) |
| *Financial Strain (Comfortable)* |  |  |  |
| Alright |  | 0.34 (-0.70 to 1.38) | 0.70 (-0.38 to 1.77) |
| Getting by |  | 1.69 ( 0.47 to 2.92) | 2.08 ( 0.81 to 3.34) |
| Quite difficult |  | 3.01 ( 1.24 to 4.78) | 3.55 ( 1.78 to 5.33) |
| Very difficult |  | 3.60 ( 1.06 to 6.14) | 4.31 ( 1.67 to 6.95) |
| *Educational and employment status (In education)* |  |  |  |
| Employment and training |  | -0.94 (-2.34 to 0.47) | -1.12 (-2.54 to 0.31) |
| NEET |  | 0.54 (-0.55 to 1.64) | 0.73 (-0.40 to 1.85) |
| *Two parent house (no)* |  |  |  |
| Yes |  |  | 0.49 (-0.54 to 1.51) |
| *Working parent (yes)* |  |  |  |
| No |  |  | -0.72 (-1.61 to 0.18) |
| *Housing Tenure (Owner)* |  |  |  |
| Rent and other |  |  | -0.58 (-1.52 to 0.37) |
| *Other children in household (none )* |  |  |  |
| One or two |  |  | -0.39 (-1.28 to 0.50) |
| Three or more |  |  | -0.96 (-2.66 to 0.75) |
| *Parental Education (Degree)* |  |  |  |
| School level |  |  | -0.55 (-1.63 to 0.53) |
| No qualification |  |  | -0.97 (-2.09 to 0.15) |
| Other not classifiable |  |  | 1.65 (-0.27 to 3.56) |
| *Ethnicity (White British)* |  |  |  |
| Other |  |  | -0.26 (-1.13 to 0.62) |
| *UK country dummy (Scotland)* |  |  |  |
| Wales |  |  | -0.84 (-2.90 to 1.22) |
| Northern Ireland |  |  | -0.33 (-2.33 to 1.67) |
| *Difference-in-differences* |  |  |  |
| Transition period | 4.20 ( 1.12 to 7.28) | 4.77 ( 1.75 to 7.80) | 5.01 ( 1.80 to 8.22) |
| Post EMA | 2.89 ( 0.67 to 5.11) | 3.16 ( 0.92 to 5.40) | 3.09 ( 0.89 to 5.29) |

Supplementary table 2: All regression coefficients for models 1, 2, 3, predicting MCS for young people in the bottom 10% of incomes in Understanding Society.

| Variable (ref category) | Model 1 | Model 2 | Model 3 |
| --- | --- | --- | --- |
|  | Coef (95% CI) | Coef (95% CI) | Coef (95% CI) |
| Intercept | 52.08 ( 47.71 to 56.44) | 54.62 ( 50.08 to 59.17) | 51.74 ( 46.48 to 57.00) |
| Area of UK - RUK |  |  |  |
| England | 2.02 ( -1.22 to 5.26) | 2.09 ( -1.26 to 5.44) | 1.87 ( -1.91 to 5.65) |
| *EMA Policy Period (EMA Period)* |  |  |  |
| Transition | 2.54 ( -1.39 to 6.46) | 3.30 ( -0.56 to 7.16) | 2.38 ( -1.33 to 6.08) |
| Post EMA | -0.91 ( -4.65 to 2.83) | -1.03 ( -4.87 to 2.81) | -1.61 ( -5.22 to 2.01) |
| *Age start school year (16)* |  |  |  |
| 17 | -0.71 ( -2.35 to 0.94) | -0.44 ( -2.11 to 1.22) | -0.58 ( -2.25 to 1.09) |
| *Gender (Male)* |  |  |  |
| Female | -3.50 ( -5.12 to -1.87) | -3.88 ( -5.51 to -2.26) | -4.08 ( -5.73 to -2.44) |
| *Month of interview (January)* |  |  |  |
| February | -1.07 ( -5.02 to 2.88) | -1.92 ( -5.83 to 1.98) | -1.94 ( -5.81 to 1.94) |
| March | -1.42 ( -5.96 to 3.11) | -2.24 ( -6.78 to 2.31) | -2.69 ( -7.41 to 2.03) |
| April | -2.80 ( -6.27 to 0.67) | -3.30 ( -6.78 to 0.18) | -3.16 ( -6.70 to 0.38) |
| May | -4.39 ( -8.59 to -0.18) | -4.08 ( -8.33 to 0.17) | -4.46 ( -8.65 to -0.28) |
| June | -0.53 ( -4.86 to 3.80) | -0.99 ( -5.36 to 3.38) | -1.26 ( -5.68 to 3.16) |
| July | -2.51 ( -6.95 to 1.93) | -3.36 ( -7.79 to 1.08) | -3.27 ( -7.62 to 1.07) |
| August | -3.24 ( -7.43 to 0.94) | -4.42 ( -8.56 to -0.27) | -4.53 ( -8.80 to -0.26) |
| September | -0.38 ( -4.11 to 3.35) | -1.35 ( -5.03 to 2.33) | -1.59 ( -5.26 to 2.09) |
| October | 2.18 ( -1.35 to 5.70) | 1.77 ( -1.70 to 5.25) | 1.43 ( -2.18 to 5.04) |
| November | -0.16 ( -4.17 to 3.84) | -0.60 ( -4.73 to 3.54) | -1.26 ( -5.34 to 2.81) |
| December | -1.81 ( -6.29 to 2.68) | -2.75 ( -7.27 to 1.76) | -2.72 ( -7.46 to 2.01) |
| *Financial Strain (Comfortable)* |  |  |  |
| Alright |  | -0.33 ( -2.46 to 1.80) | -1.28 ( -3.44 to 0.87) |
| Getting by |  | -3.10 ( -5.53 to -0.68) | -4.26 ( -6.78 to -1.74) |
| Quite difficult |  | -3.81 ( -8.13 to 0.50) | -5.28 ( -9.52 to -1.04) |
| Very difficult |  | -7.01 (-12.02 to -1.99) | -8.86 (-13.82 to -3.89) |
| *Educational and employment status (In education)* |  |  |  |
| Employment and training |  | 1.90 ( -0.68 to 4.48) | 2.01 ( -0.61 to 4.64) |
| NEET |  | -1.28 ( -3.51 to 0.95) | -1.75 ( -4.12 to 0.62) |
| *Two parent house (no)* |  |  |  |
| Yes |  |  | 0.04 ( -2.01 to 2.09) |
| *Working parent (yes)* |  |  |  |
| No |  |  | 2.83 ( 1.04 to 4.61) |
| *Housing Tenure (Owner)* |  |  |  |
| Rent and other |  |  | 1.01 ( -0.85 to 2.86) |
| *Other children in household (none )* |  |  |  |
| One or two |  |  | 1.06 ( -0.72 to 2.83) |
| Three or more |  |  | 0.24 ( -4.75 to 5.22) |
| *Parental Education (Degree)* |  |  |  |
| School level |  |  | 1.80 ( -0.47 to 4.07) |
| No qualification |  |  | 3.44 ( 0.99 to 5.89) |
| Other not classifiable |  |  | -2.35 ( -5.85 to 1.15) |
| *Ethnicity (White British)* |  |  |  |
| Other |  |  | 0.81 ( -1.02 to 2.65) |
| *UK country dummy (Scotland)* |  |  |  |
| Wales |  |  | 1.96 ( -2.04 to 5.96) |
| Northern Ireland |  |  | 0.39 ( -3.59 to 4.38) |
| *Difference-in-differences* |  |  |  |
| Transition period | -7.45 (-12.27 to -2.64) | -8.64 (-13.34 to -3.94) | -8.06 (-12.80 to -3.31) |
| Post EMA | -3.79 ( -7.95 to 0.37) | -4.37 ( -8.60 to -0.14) | -3.59 ( -7.63 to 0.44) |

Supplementary table 3: All regression coefficients for models 1, 2, 3, predicting PCS for young people in the bottom 10% of incomes in Understanding Society.

| Variable (ref category) | Model 1 | Model 2 | Model 3 |
| --- | --- | --- | --- |
|  | Coef (95% CI) | Coef (95% CI) | Coef (95% CI) |
| Intercept | 52.93 (50.20 to 55.67) | 54.65 (51.82 to 57.48) | 57.50 (54.23 to 60.78) |
| Area of UK - RUK |  |  |  |
| England | 1.50 (-0.96 to 3.97) | 1.34 (-1.07 to 3.75)5 | 0.89 (-1.84 to 3.62) |
| *EMA Policy Period (EMA Period)* |  |  |  |
| Transition | -0.54 (-4.05 to 2.98) | -0.02 (-3.39 to 3.35) | -0.03 (-3.44 to 3.38) |
| Post EMA | 2.94 ( 0.20 to 5.68) | 2.16 (-0.50 to 4.82) | 1.65 (-1.03 to 4.33) |
| *Age start school year (16)* |  |  |  |
| 17 | -0.21 (-1.16 to 0.73) | 0.00 (-0.97 to 0.97) | -0.07 (-1.06 to 0.91) |
| *Gender (Male)* |  |  |  |
| Female | -0.70 (-1.63 to 0.23) | -0.73 (-1.67 to 0.21) | -0.67 (-1.65 to 0.30) |
| *Month of interview (January)* |  |  |  |
| February | -0.33 (-2.63 to 1.97) | -0.20 (-2.58 to 2.18) | 0.33 (-2.04 to 2.69) |
| March | 1.30 (-0.93 to 3.53) | 1.32 (-0.97 to 3.61) | 1.72 (-0.61 to 4.06) |
| April | -1.51 (-3.55 to 0.53) | -1.36 (-3.33 to 0.61) | -1.47 (-3.48 to 0.54) |
| May | -1.16 (-3.46 to 1.14) | -0.33 (-2.71 to 2.05) | 0.27 (-2.23 to 2.77) |
| June | -1.10 (-3.37 to 1.17) | -0.84 (-3.09 to 1.40) | -0.44 (-2.76 to 1.88) |
| July | 1.33 (-0.52 to 3.17) | 1.58 (-0.28 to 3.44) | 1.77 (-0.11 to 3.64) |
| August | 0.09 (-2.17 to 2.36) | 0.44 (-1.86 to 2.73) | 0.63 (-1.68 to 2.94) |
| September | -1.14 (-3.07 to 0.80) | -1.23 (-3.24 to 0.79) | -1.02 (-3.03 to 0.98) |
| October | -1.42 (-3.70 to 0.86) | -1.83 (-4.02 to 0.35) | -1.59 (-3.75 to 0.56) |
| November | 0.53 (-1.27 to 2.33) | 0.87 (-0.99 to 2.72) | 1.36 (-0.54 to 3.26) |
| December | -0.19 (-2.28 to 1.90) | -0.15 (-2.30 to 2.01) | -0.05 (-2.34 to 2.24) |
| *Financial Strain (Comfortable)* |  |  |  |
| Alright |  | -0.93 (-2.05 to 0.18) | -0.71 (-1.83 to 0.41) |
| Getting by |  | -1.49 (-2.85 to -0.13) | -1.40 (-2.77 to -0.03) |
| Quite difficult |  | -1.58 (-3.67 to 0.50) | -1.20 (-3.35 to 0.94) |
| Very difficult |  | -3.63 (-6.65 to -0.60) | -3.28 (-6.52 to -0.05) |
| *Educational and employment status (In education)* |  |  |  |
| Employment and training |  | -0.79 (-2.26 to 0.67) | -0.78 (-2.29 to 0.73) |
| NEET |  | -2.07 (-3.42 to -0.73) | -1.97 (-3.40 to -0.55) |
| *Two parent house (no)* |  |  |  |
| Yes |  |  | -0.65 (-1.79 to 0.49) |
| *Working parent (yes)* |  |  |  |
| No |  |  | -1.37 (-2.42 to -0.32) |
| *Housing Tenure (Owner)* |  |  |  |
| Rent and other |  |  | -0.29 (-1.34 to 0.77) |
| *Other children in household (none )* |  |  |  |
| One or two |  |  | -0.49 (-1.56 to 0.59) |
| Three or more |  |  | 2.05 ( 0.42 to 3.69) |
| *Parental Education (Degree)* |  |  |  |
| School level |  |  | -1.29 (-2.49 to -0.09) |
| No qualification |  |  | -2.44 (-3.80 to -1.08) |
| Other not classifiable |  |  | -0.48 (-2.46 to 1.50) |
| *Ethnicity (White British)* |  |  |  |
| Other |  |  | -0.22 (-1.34 to 0.91) |
| *UK country dummy (Scotland)* |  |  |  |
| Wales |  |  | -1.49 (-4.46 to 1.48) |
| Northern Ireland |  |  | -0.25 (-2.59 to 2.09) |
| *Difference-in-differences* |  |  |  |
| Transition period | 2.04 (-1.80 to 5.88) | 1.26 (-2.45 to 4.96) | 0.81 (-2.96 to 4.58) |
| Post EMA | -2.13 (-5.09 to 0.83) | -1.82 (-4.71 to 1.07) | -1.84 (-4.72 to 1.05) |
|  |  |  |  |

Supplementary table 4: All regression coefficients for models 1, 2, 3, predicting life satisfaction for young people in the bottom 10% of incomes in Understanding Society.

| Variable (ref category) | Model 1 | Model 2 | Model 3 |
| --- | --- | --- | --- |
|  | Coef (95% CI) | Coef (95% CI) | Coef (95% CI) |
| Intercept | 5.16 ( 4.59 to 5.73) | 5.86 ( 5.27 to 6.46) | 5.89 ( 5.21 to 6.56) |
| Area of UK – RUK |  |  |  |
| England | 0.33 (-0.13 to 0.79) | 0.32 (-0.14 to 0.79) | 0.15 (-0.35 to 0.65) |
| *EMA Policy Period (EMA Period)* |  |  |  |
| Transition | 0.30 (-0.44 to 1.04) | 0.40 (-0.25 to 1.05) | 0.24 (-0.44 to 0.91) |
| Post EMA | 0.20 (-0.29 to 0.70) | 0.10 (-0.41 to 0.60) | 0.02 (-0.47 to 0.50) |
| *Age start school year (16)* |  |  |  |
| 17 | -0.01 (-0.22 to 0.20) | 0.10 (-0.10 to 0.30) | 0.10 (-0.11 to 0.31) |
| *Gender (Male)* |  |  |  |
| Female | 0.03 (-0.18 to 0.23) | -0.06 (-0.25 to 0.14) | -0.08 (-0.29 to 0.12) |
| *Month of interview (January)* |  |  |  |
| February | 0.04 (-0.48 to 0.55) | -0.05 (-0.56 to 0.46) | -0.03 (-0.55 to 0.50) |
| March | 0.33 (-0.17 to 0.82) | 0.26 (-0.23 to 0.74) | 0.30 (-0.20 to 0.80) |
| April | -0.13 (-0.60 to 0.35) | -0.14 (-0.61 to 0.34) | -0.15 (-0.63 to 0.33) |
| May | -0.17 (-0.68 to 0.34) | -0.13 (-0.62 to 0.37) | -0.07 (-0.57 to 0.44) |
| June | -0.11 (-0.60 to 0.39) | -0.09 (-0.57 to 0.38) | -0.11 (-0.59 to 0.38) |
| July | -0.17 (-0.74 to 0.39) | -0.23 (-0.77 to 0.30) | -0.23 (-0.77 to 0.31) |
| August | -0.03 (-0.52 to 0.46) | -0.07 (-0.55 to 0.41) | -0.10 (-0.60 to 0.41) |
| September | -0.01 (-0.48 to 0.46) | -0.12 (-0.59 to 0.35) | -0.14 (-0.62 to 0.34) |
| October | 0.28 (-0.21 to 0.76) | 0.20 (-0.28 to 0.67) | 0.23 (-0.26 to 0.72) |
| November | -0.03 (-0.50 to 0.44) | -0.03 (-0.49 to 0.42) | -0.07 (-0.55 to 0.40) |
| December | -0.02 (-0.54 to 0.49) | -0.13 (-0.63 to 0.38) | -0.19 (-0.73 to 0.35) |
| *Financial Strain (Comfortable)* |  |  |  |
| Alright |  | -0.43 (-0.68 to -0.18) | -0.51 (-0.77 to -0.25) |
| Getting by |  | -0.83 (-1.13 to -0.54) | -0.89 (-1.20 to -0.58) |
| Quite difficult |  | -1.30 (-1.74 to -0.86) | -1.35 (-1.81 to -0.90) |
| Very difficult |  | -1.52 (-2.11 to -0.93) | -1.73 (-2.34 to -1.12) |
| *Educational and employment status (In education)* | |  |  |
| Employment and training |  | 0.20 (-0.11 to 0.51) | 0.18 (-0.16 to 0.51) |
| NEET |  | -0.19 (-0.47 to 0.10) | -0.20 (-0.51 to 0.10) |
| *Two parent house (no)* |  |  |  |
| Yes |  |  | -0.09 (-0.34 to 0.16) |
| *Working parent (yes)* |  |  |  |
| No |  |  | 0.31 ( 0.09 to 0.53) |
| *Housing Tenure (Owner)* |  |  |  |
| Rent and other |  |  | 0.01 (-0.22 to 0.24) |
| *Other children in household (none )* |  |  |  |
| One or two |  |  | -0.05 (-0.27 to 0.18) |
| Three or more |  |  | -0.03 (-0.45 to 0.38) |
| *Parental Education (Degree)* |  |  |  |
| School level |  |  | 0.18 (-0.09 to 0.46) |
| No qualification |  |  | 0.14 (-0.16 to 0.44) |
| Other not classifiable |  |  | -0.39 (-0.83 to 0.04) |
| *Ethnicity (White British)* |  |  |  |
| Other |  |  | -0.06 (-0.30 to 0.18) |
| *UK country dummy (Scotland)* |  |  |  |
| Wales |  |  | -0.18 (-0.67 to 0.30) |
| Northern Ireland |  |  | -0.02 (-0.56 to 0.51) |
| *Difference-in-differences* |  |  |  |
| Transition period | -0.81 (-1.65 to 0.03) | -0.99 (-1.74 to -0.23) | -0.75 (-1.54 to 0.04) |
| Post EMA | -0.47 (-1.03 to 0.08) | -0.55 (-1.11 to 0.02) | -0.42 (-0.97 to 0.13) |
|  |  |  |  |

Supplementary table 5: Difference-in-differences estimates^1^ for the associations with changing EMA policy period in England on GHQ12, MCS, PCS for young people living in households in the lowest10% of income stratified by gender.

|  | Male |  | Female |
| --- | --- | --- | --- |
|  | Coef (95% CI) |  | Coef (95% CI) |
| *GHQ12* |  |  |  |
| Transition Period | 5.49 (1.72 to 9.27) |  | 2.70 (-2.38 to 7.78) |
| Post EMA | 1.62 (-1.59 to 4.83) |  | 4.14 (1.02 to 7.25) |
| *MCS* |  |  |  |
| Transition Period | -10.76 (-17.07 to -4.45) |  | -4.45 (-11.93 to 3.02) |
| Post EMA | -3.97 (-10.17 to 2.22) |  | -3.96 (-9.81 to 1.89) |
| *PCS* |  |  |  |
| Transition Period | 0.96 (-3.94 to 5.86) |  | 3.61 (-2.39 to 9.60) |
| Post EMA | -4.66 (-8.83 to -0.49) |  | 0.26 (-3.96 to 4.48) |
| *Life Satisfaction* |  |  |  |
| Transition Period | -1.90 (-2.90 to -0.90) |  | 0.41 (-0.83 to 1.65) |
| Post EMA | -0.65 (-1.49 to 0.20) |  | -0.27 (-1.04 to 0.49) |

1 An interaction term between UK country and EMA period. Models also include main effects for UK area, EMA policy period, Age at start of school year, gender, Month of interview as well as the difference- in-difference estimate.

Supplementary table 6: Difference-in-differences estimates for the associations with changing EMA policy period in England on GHQ12, MCS, PCS for young people living in households in the lowest10% of income stratified by ethnicity.

|  | White British |  | Other |
| --- | --- | --- | --- |
|  | Coef (95% CI) |  | Coef (95% CI) |
| *GHQ12* |  |  |  |
| Transition Period | 4.23 (0.55 to 7.91) |  | 5.22 (-2.76 to 13.20) |
| Post EMA | 3.54 (0.99 to 6.10) |  | 1.83 (-4.01 to 7.66) |
| *MCS* |  |  |  |
| Transition Period | -5.05 (-10.54 to 0.45) |  | -15.00 (-26.32 to -3.69) |
| Post EMA | -4.52 (-9.17 to 0.13) |  | -1.18 (-9.67 to 7.32) |
| *PCS* |  |  |  |
| Transition Period | 1.60 (-2.84 to 6.04) |  | 1.52 (-6.16 to 9.19) |
| English Post EMA | -1.93 (-5.27 to 1.41) |  | -1.98 (-8.58 to 4.62) |
| *Life Satisfaction* |  |  |  |
| Transition Period | -0.43 (-1.40 to 0.55) |  | -0.87 (-2.74 to 1.00) |
| Post EMA | -0.53 (-1.15 to 0.08) |  | 0.17 (-1.13 to 1.47) |

1 An interaction term between UK country and EMA period. Models also include main effects for UK area, EMA policy period, Age at start of school year, gender, Month of interview as well as the difference-in-difference estimate.

Supplementary table 7: Difference-in-differences estimates for the associations with changing EMA policy period in England on GHQ12, MCS, PCS for young people living in households in the lowest10% of income stratified by age.

|  | Age 16 |  | Age 17 |
| --- | --- | --- | --- |
|  | Coef (95% CI) |  | Coef (95% CI) |
| *GHQ12* |  |  |  |
| Transition Period | 6.19 (2.33 to 10.05) |  | 1.24 (-3.40 to 5.87) |
| Post EMA | 3.18 (0.08 to 6.27) |  | 2.41 (-0.98 to 5.80) |
| *MCS* |  |  |  |
| Transition Period | -6.70 (-13.72 to 0.33) |  | -7.76 (-14.76 to -0.77) |
| Post EMA | -1.46 (-6.70 to 3.78) |  | -6.29 (-13.01 to 0.43) |
| *PCS* |  |  |  |
| Transition Period | 3.85 (-0.90 to 8.60) |  | 0.09 (-6.02 to 6.19) |
| Post EMA | -1.67 (-4.97 to 1.62) |  | -2.45 (-7.45 to 2.55) |
| *Life Satisfaction* |  |  |  |
| Transition Period | -0.82 (-2.20 to 0.56) |  | -0.68 (-1.64 to 0.28) |
| Post EMA | -0.62 (-1.46 to 0.22) |  | -0.34 (-1.11 to 0.44) |

1 An interaction term between UK country and EMA period. Models also include main effects for UK area, EMA policy period, Age at start of school year, gender, Month of interview as well as the difference-in- difference estimate.

## Figures

Supplementary Figure 1: flow chart for derivation of analytic sample

``

Excluded not in bottom 10% of income

N = 10,576

Has an outcome measure

N = 11,904

Interview data outside academic years 2009 to 2018

N = 56

Missing residency data

N = 4

Not eligible due to having valid academic year weights

N = 1,284,428

Data available on country of residence and resident in England, Scotland Wales and Northern Ireland

N = 12,103

Lives with foster parent

N = 42

Aged 16 or 17 on the 1^st^ Sept of Academic year

N = 12,107

Total number of observation records in Understanding Society

N = 1,654,323

Has valid weight for academic years 2009 to 2018

N = 369,895

Excluded no outcome measure

N = 101

Outside study age range

N = 357,88

Not a care leaver

N = 12,061

Final analytic sample

N = 1,328

Date of interview in Academic years 2009 to 2018

N = 11,090

Supplementary Figure 2: Predicted mental health outcomes by EMA policy period for young people in the lowest 10% of household incomes in England and the Rest of UK (RUK).


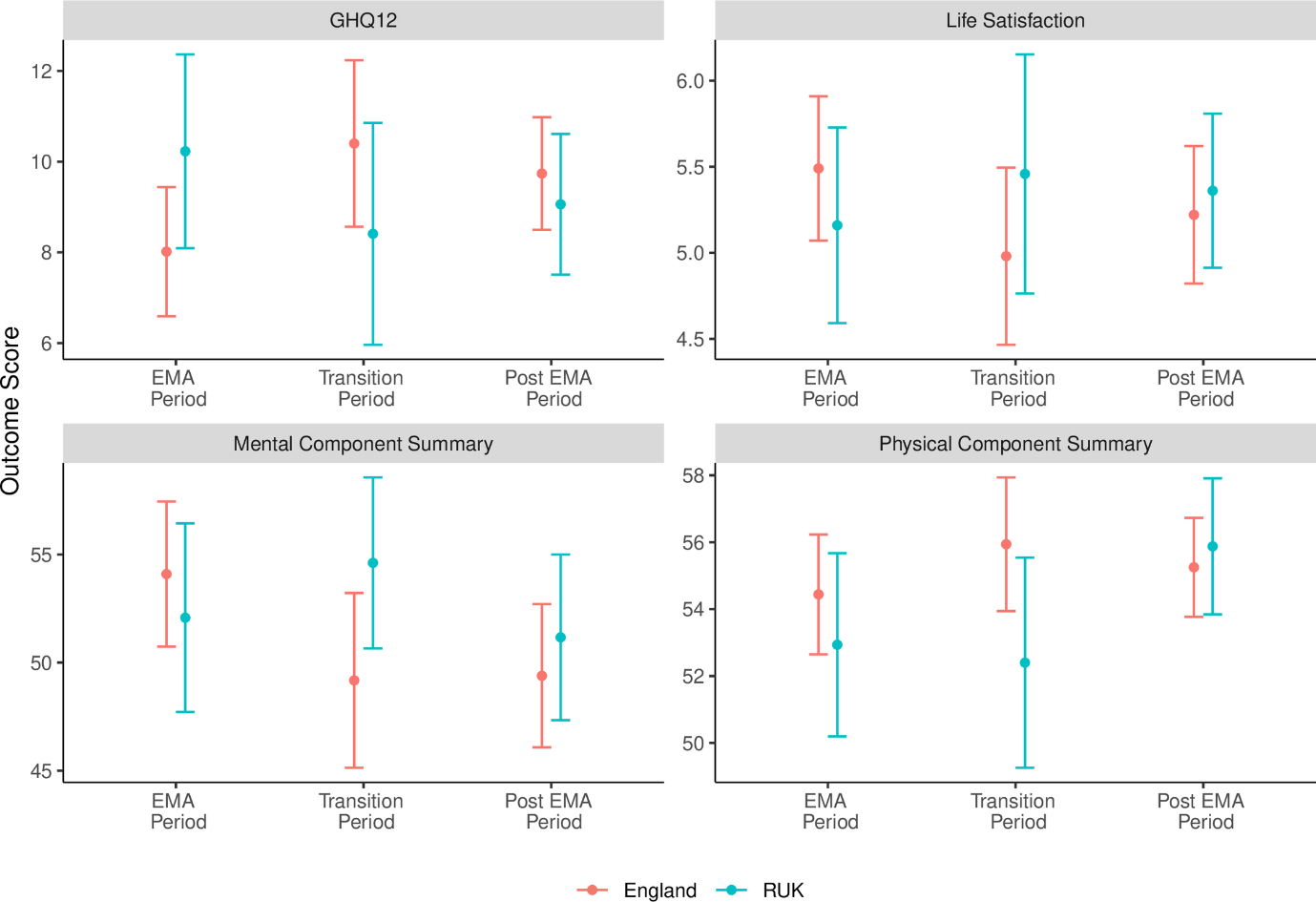


Supplementary Figure 3: GHQ12 scores over time for participants of the British Household Panel Survey who are aged 16 or 17 and in the bottom 10% for household incomes.


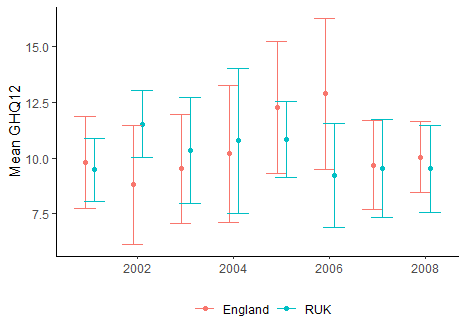

Supplement: online supplemental file 1 [file bmjph-3-1-s001.docx]
